# Understanding the exposure risk of aerosolized *Coccidioides* in a Valley fever endemic metropolis

**DOI:** 10.1038/s41598-024-51407-x

**Published:** 2024-01-15

**Authors:** W. Tanner Porter, Lalitha Gade, Parker Montfort, Joseph R. Mihaljevic, Jolene R. Bowers, Andrew Willman, Brian A. Klimowski, Bonnie J. LaFleur, Rebecca H. Sunenshine, Jennifer Collins, Guillermo Adame, Shane Brady, Kenneth K. Komatsu, Samantha Williams, Mitsuru Toda, Tom Chiller, Anastasia P. Litvintseva, David M. Engelthaler

**Affiliations:** 1 Pathogen & Microbiome Division, Translational Genomics Research Institute, Flagstaff, AZ USA; 2 Mycotic Diseases Branch, Centers for Disease Control and Prevention, Atlanta, GA USA; 3 School of Informatics, Computing, and Cyber Systems, Northern Arizona University, Flagstaff, AZ USA; 4 Department of Homeland Security, Phoenix, AZ USA; 5 National Weather Service, Flagstaff, AZ USA; 6 College of Pharmacy, The University of Arizona, Phoenix, AZ USA; 7 Maricopa County Department of Public Health, Phoenix, AZ USA; 8 Arizona Department of Health Services, Phoenix, AZ USA

**Keywords:** Ecology, Ecological epidemiology, Infectious diseases, Fungal infection

## Abstract

*Coccidioides* is the fungal causative agent of Valley fever, a primarily pulmonary disease caused by inhalation of fungal arthroconidia, or spores. Although *Coccidioides* has been an established pathogen for 120 years and is responsible for hundreds of thousands of infections per year, little is known about when and where infectious *Coccidioides* arthroconidia are present within the ambient air in endemic regions. Long-term air sampling programs provide a means to investigate these characteristics across space and time. Here we present data from > 18 months of collections from 11 air sampling sites across the Phoenix, Arizona, metropolitan area. Overall, prevalence was highly variable across space and time with no obvious spatial or temporal correlations. Several high prevalence periods were identified at select sites, with no obvious spatial or temporal associations. Comparing these data with weather and environmental factor data, wind gusts and temperature were positively associated with *Coccidioides* detection, while soil moisture was negatively associated with *Coccidioides* detection. These results provide critical insights into the frequency and distribution of airborne arthroconidia and the associated risk of inhalation and potential disease that is present across space and time in a highly endemic locale.

## Introduction

The changing nature of modern climates and landscapes requires a closer investigation of pathogenic environmental microbes. An oft-referenced pathogen tied to climate change is *Coccidioides*, the causative agent of coccidioidomycosis, or Valley fever. These dimorphic soil-dwelling fungi within the Onygenales order and are represented by two phylogenetically distinct species, *C. posadasii* and *C. immitis*^1,2^*. C. immitis* is found in desert regions in central and southern California and Baja California, as well as isolated locations in south-central Washington state, while *C. posadasii* is found in the thermic and arid desert regions of Arizona, Nevada, New Mexico, Texas, Mexico, and Central and South America^3^. Exposure to *Coccidioides* is estimated to result in at least 150,000 cases each year in the United States, with the vast majority reported from Arizona and California^3,4^; however, only a fraction (10,000–20,000 cases) are officially reported to public health agencies^3,4^. This under-reporting is thought to be the result of limited medical awareness, frequent misdiagnosis, lack of care seeking, testing and diagnostic challenges, and variations in reporting guidelines^3–6^. Most individuals become infected by inhaling airborne infectious fungal spores or arthroconidia, yet the factors that facilitate and maintain aerosolization of arthroconidia are poorly understood. It is thought that the *Coccidioides* lifecycle and ecology along with weather and anthropogenic factors drive environmental aerosolization.

*Coccidioides* fungi have a complex lifecycle that includes two distinct morphologies. The first is the saprophytic cycle that occurs within soil where mycelia develop before forming infectious arthroconidia. The second is the parasitic cycle which occurs when arthroconidia change into immature spherules and develop numerous endospores which can colonize tissues^1^. The role of the mammalian hosts has been an area of recent interest in the mammalian-driver hypothesis^7,8^, in which small mammalian hosts are the drivers of this system, allowing high burdens of endospores within a host to colonize environmental soils and transition to a saprophytic cycle once the host dies from the disease, before infecting a successive host^7^. In this, *Coccidioides* may be an endozoan organism that does not necessarily kill the host, but once the host dies, the carcass provides the needed resources for an extended saprophytic life cycle within the environment^8^. However, once the fungus has transitioned to the saprophytic cycle, subsequent infections are the result of arthroconidia production and further spread to naïve susceptible hosts via soil disturbance and inhalation of disarticulated conidia^1,7,8^. It has also been shown that *Coccidioides* can persist in soil for at least 6 years^9^ and perhaps for thousands of years or more under ideal soil and microclimate conditions^10^. Human infections are primarily driven by inhalation of aerosolized arthroconidia^11^ and, based on coccidioidin skin test surveys, exposure is common in individuals inhabiting endemic regions^12,13^. Although research has focused on characterizing the ecology and distribution of *Coccidioides* within the soil environment^9,14–16^, limited research has characterized the distribution or abundance of arthroconidia within the air^17,18^, restricting our understanding of drivers of arthroconidia aerosolization and human exposure risk.

As the burden of Valley fever cases continues to increase^4^ and climate shifts continue to shape the world around us, it is essential to identify the environmental factors that facilitate the growth of the fungus and dispersal of the arthroconidia within the air to better model the risk of contracting this disease in endemic regions. However, these dynamics are complex, and are likely driven by many factors that vary across space and time. For example, at a coarse spatial scale (e.g., hundreds of kms), general climatic variables (e.g., average temperature, precipitation, etc.) influence the area that *Coccidioides* could survive in or the suitable climatic niche of *Coccidioides*. At an intermediate spatial scale (e.g., ~ 10–100 kms), seasonal weather events and weather patterns (with anomalous precipitation, temperature, or winds) influence areas or periods of time that may promote higher abundances of *Coccidioides* and/or its mammalian hosts^19^. At a fine spatial scale (e.g., 0.1–10 kms), anthropogenic impacts (e.g., agriculture and construction)^20,21^, highly localized weather events (e.g., rain events and wind event), and natural disasters (e.g., earthquakes, wildfires, etc.)^21,22^ increase the amount of soil disturbance allowing for potential increases in arthroconidia aerosolization. However, endemic presence of *Coccidioides* is dependent on *Coccidioides* being introduced into the soil (i.e., infected mammal dies below, or is buried in, the soil) and producing arthroconidia within a localized environment^10^, a phenomenon that occurs at the smallest spatial scale (e.g., a few meters). Previous soil sampling has shown that *Coccidioides* presence is one of the most challenging dynamics to predict, as the distribution is generally not consistent across space and colonization is often a stochastic event^1,8,12^.

Previous work has explored the relationship between possible environmental factors and Valley fever cases and *Coccidioides* exposure using statistical methods to model the number of human clinical cases across space and time using weather, dust storm, and airborne particulate datasets (e.g., PM_10_ and PM_2.5_) (Table 1). However, these methods are limited by variability in time between arthroconidia exposure (inhalation) and diagnosis, underreporting of clinical cases, and lack of knowledge regarding the exposure location, which challenges the associations that can be identified. Although these attempts have limitations, several valuable trends have been explored and characterized (Table 1). Most notably, these analyses have identified increases in Valley fever cases related to increased precipitation, allowing for hyphal growth, followed by hot and dry periods, facilitating arthroconidia formation and aerosolization. In addition, previous analyses focusing on the impact of dust storms on clinical cases have been inconclusive with contrasting findings.

**Table 1:** Previously published models linking weather or other covariates to Valley fever incidence or *Coccidioides* exposure.

Area of interest	Years	Model type	Response variable	Weather predictors considered	Other variables considered	Significant weather predictors	Significant other variables	Author conclusions	Citation
Maricopa, AZ	Aug 2015, Oct 2016, Fall 2017, Jan 2018–June 2019	Univariate binomial mixed effects model	Daily *Coccidioides* presence absence data	Wind speed, temperature, visibility, precipitation	Soil moisture, PM_10_	Wind speed	None	*Coccidioides* aerosolization increases in response to high gusts, high temps and low soil moisture	This study
California (14 counties)	2000–2020	Multiple (ensemble)	Clinical cases	Temperature, precipitation	Soil texture, elevation, % impervious surface	Lagged precipitation, lagged temperature	Dependent on model	Drought can decrease Valley fever cases in the short term but increase cases in the years following the drought conditions	^19^
Maricopa, Pima, Pinal Counties, AZ	2013–2018	General additive model (time series)	Clinical cases	Wind speed, mean max temperature, and total monthly precipitation with 2-month lag on all variables	PM_10_ with 2-month lag	Mean max temp, lagged precipitation, wind speed, precipitation	Lagged PM_10_	Lagged PM_10_ within the winter months had the largest impact on Valley fever cases	^20^
Maricopa County, AZ & Kern County, CA	2006–2020	Superposed epoch analysis	Clinical cases	None	Dust storm data	None	None	No indication of an increase in Valley fever cases following dust storm activity	^21^
San Joaquin Valley, CA and southcentral, AZ	2000–2015	Linear and nonlinear regression	Clinical cases	Air temperature, precipitation	Soil moisture, dust concentration, NDVI, cropland area	Not applicable	Not applicable	Air temperature, precipitation, soil moisture, dust concentration, NDVI, and cropland area could be significant covariates for a predictive model	^22^
Maricopa, Pima Counties, AZ	2001–2011	Correlation Analyses	Clinical cases	Precipitation	PM_10_, PM_2.5_, dust number, dust frequency	Not applicable	Not applicable	Dust frequency was correlated but does not explain all variability in Valley fever cases	^23^
Maricopa, Pima Counties, AZ	1995–2006	Correlational analyses, regression	Clinical cases	None	NDVI	None	NDVI	Inverse relationship between NDVI and Valley fever cases	^24^
Kern County, CA	1995–2003	Generalized auto regressive moving average model	Clinical cases (weekly)	Temperature, precipitation, wind speed	None	None	None	Relationship between weather parameters and Valley fever case fluctuations are weak	^25^
Kern County, CA	1980–2002	Univariate and	Clinical cases (monthly)	Temperature, precipitation, wind speed	None	Temperature, precipitation, wind speed	None	Relationship between weather parameters and Valley fever case fluctuations are weak	^26^
Pima County, AZ	1992–2003	Linear regression	Clinical cases	Precipitation	PM_10_	Precipitation (lagged)	PM_10_ (current)	Important role of precipitation with a 1.5–2-year lag prior to exposure	^27^
Maricopa County, AZ	1998–2001	Poisson regression analysis	Clinical cases	Precipitation, wind speed, temperature	Building permits, Palmer Z index, Palmer drought severity index, PM_10_	Precipitation, wind, temperature	Building permits, Palmer Z index, Palmer drought severity index, PM_10_	Poisson regression identified several weather, dust, and drought covariates as significant to Valley fever incidence	^28^
Pima County, AZ	1948–1998	Linear regression and composite analysis	Clinical cases	Precipitation (total, average. Max), dew point, average wind speed	Palmer drought severity index (PDSI)	Temperature and precipitation with different lags depending on month	None	Identified significant relationships with temperature and precipitation at different lag times corresponding to the ecology of *Coccidioides*	^29^

Unlike clinical case data, air surveillance for airborne *Coccidioides* provides a method to more directly assess Valley fever exposure risk that is not subject to the challenges associated with clinical cases and more directly measures prevalence of aerosolized *Coccidioides* in a highly endemic region^17^. A careful, comprehensive collection of air filter data could provide a means to investigate factors that facilitate and maintain arthroconidia aerosolization, a key area of research as the public health community takes a holistic view of Valley fever. Here, we describe results and analysis from a long-term air surveillance campaign in the greater Phoenix, AZ, metropolitan area. Through this campaign, we aimed to detect aerosolized *Coccidioides* across an endemic metropolitan region over an 18-month period, as well as identify factors that influence aerosolization, including daily local weather covariates, dust storms, and land cover.

## Results

### *Coccidioides* air surveillance

**Figure 1:**
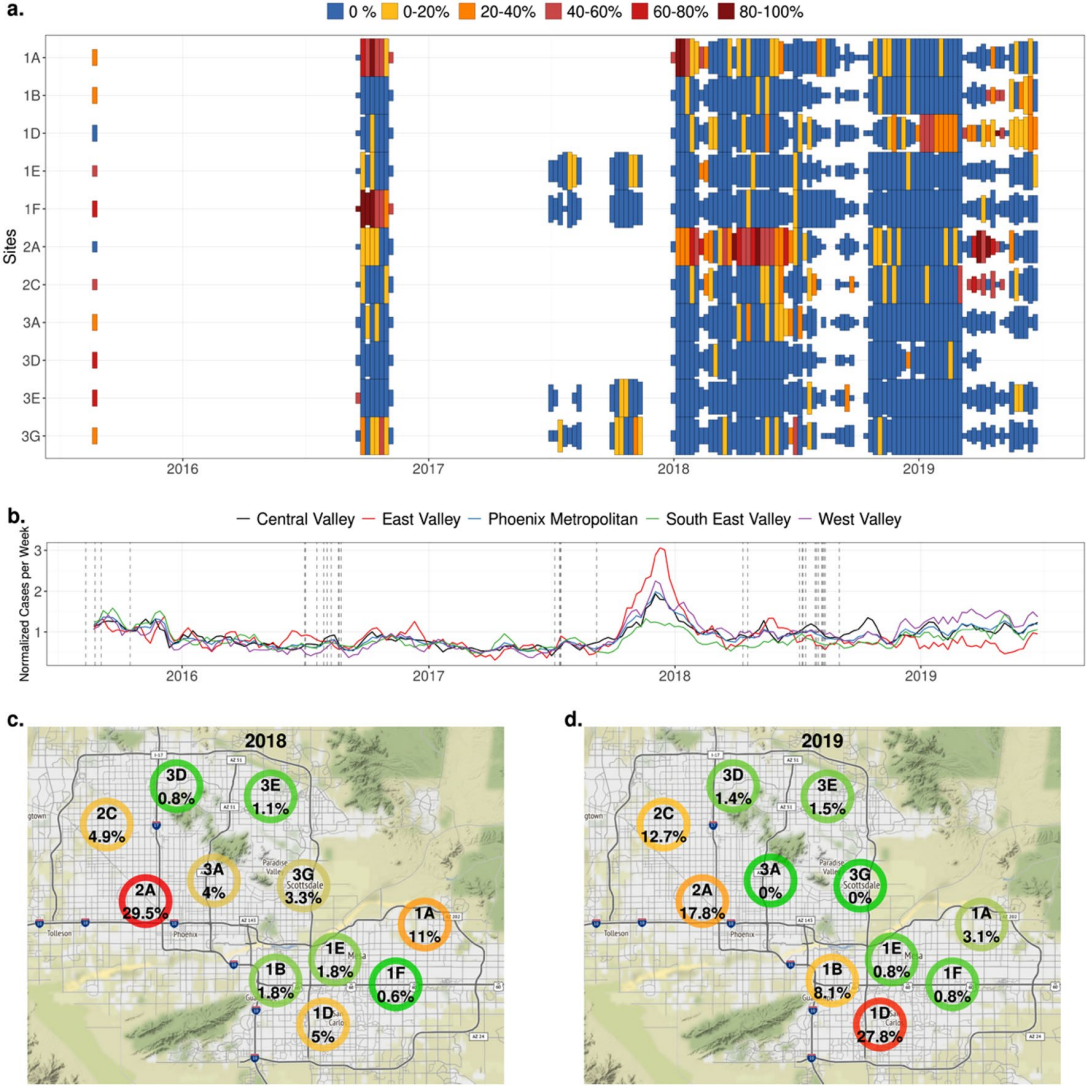
(**a**) Weekly prevalence of *Coccidioides* in ambient air sampling across the Phoenix metropolitan area, 2015–2019. The height of the tile represents the number of filter samples collected within the week (min = 1 day, max = 7 day). The color corresponds to the percentage of filter samples that were positive. (**b**) Normalized number of reported human Valley fever cases per week in greater Phoenix metropolitan area, AZ, 2015–2019. All cases in the greater Phoenix metropolitan area are included as the Phoenix metropolitan with finer scale aggregation included in the West Valley, Central Valley, East Valley, and South East Valley. Dotted vertical lines represent days with dust storm events reported to the Storm Events Database of the US National Centers for Environmental Information. (**c**) Prevalence of *Coccidioides* in ambient air sampling across the greater Phoenix metropolitan area during 2018 (Jan–Dec), with green representing low prevalence, yellow representing moderate prevalence, and red representing high prevalence. (**d**) Prevalence of *Coccidioides* in ambient air sampling across the greater Phoenix metropolitan area during 2019 (Jan–June), with green representing low prevalence, yellow representing moderate prevalence, and red representing high prevalence. Maps were created in R (4.2.0)^30^ using Rstudio editor (2022.02.2)^31^ and ggmap^32^.

In total, 5243 filters from 630 days were tested for *Coccidioides* DNA across 11 sites in the greater Phoenix metropolitan area (Fig. 1a, Supplemental Table 1, Supplemental File 1). These data incorporate collections from three pilot programs and a sustained surveillance program that covered an 18-month period. During the first pilot program, samples were collected from 11 sites across 3 days in late August 2015. The second pilot program covered an 8-week period in 2016 (mid-September to early November)^17^. The third pilot program collected samples from three sites across several weeks in the latter half of 2017. The sustained surveillance program was the largest and most protracted and was conducted across 11 sites between January 2018 and June 2019.

An average of 5.45 (median: 7) filters were collected each week across all sites. Weekly *Coccidioides* prevalence (i.e., number of daily positive filters per week/total number of daily filters tested per week) varied from 0 to 100%, with a mean of 7.4% (median: 0%) across all sites (Fig. 1a). Positive *Coccidioides* weeks were often grouped over a limited duration (i.e., days to weeks) by site. *Coccidioides* was detected at a high prevalence for ~ 6 months at one site (Site 2A: January 2018–June 2018); however, several other sites had periods of sustained prevalence for several weeks (Sites 1A, 1D, 1F). Qualitatively, there was not a clear relationship between *Coccidioides* filter prevalence and clinical cases, with a sustained number of clinical cases across the Phoenix metropolitan area with the exception of an obvious and previously reported^33^ spike in cases at the end of 2017 (Fig. 1b).

Throughout 2018, the yearly site prevalence ranged from 0.6 to 29.5% (mean: 5.8%, median: 3.3%) (Fig. 1c). In 2019, yearly site prevalence ranged from 0 to 27.8% (mean: 6.7%, median: 1.5%) (Fig. 1d). The higher prevalence sites (1A,1B, 1D, 2A, and 2C) were perimeter sites, with four of the five located on the eastern or southern perimeters of the Phoenix metropolitan area. Similar to yearly *Coccidioides* site prevalence, across the 18-month study period (2018–2019), *Coccidioides* prevalence was highly variable across seasons and sites, with the highest prevalence in the spring of 2019 (11.5%, 59/452, 95% CI 9.0–14.7%) and 2018 (8.3%, 81/893, 95% CI 6.7–10.3%), however; this trend was not consistent across sites with individual sites showing variable seasonal prevalence (Supplemental Fig. 1).

### *Coccidioides* presence comparison with dust storms

Across the entire surveillance program, days with a reported dust storm had a *Coccidioides* prevalence of 5% (7/131, 95% CI 2.3–11.1%) compared with days without dust storms which had a prevalence of 7% (368/5112, 95% CI 6.5–7.9%), p = 0.52. In 2018, a total of 14 dust storms were either reported to National Oceanic & Atmospheric Administration’s (NOAA) Storm Events Database (n = 13; 9/13 verified via photographic evidence) or identified through an investigator online search (n = 1). *Coccidioides* prevalence was ~ 5% (Table 2) on days with reported dust storms, while the prevalence was ~ 6% for the filters collected within the 7-days prior to the dust storm. There was no statistically significant difference in prevalence between days with a dust storm and days without utilizing all dust storm events (p = 0.83), only NOAA reported events (p = 0.61), or utilizing only investigator verified events (p = 0.76) (Table 2).

**Table 2:** *Coccidioides* filter prevalence in 2018 on days with dust storms and the proceeding 7-days across all identified dust storms, all NOAA reported dust storms, and all investigator verified events.

Dust storm definition	N dust storms	*Coccidioides* filter prevalence on days with dust storms [%(n/total, 95% CI)]	Average *Coccidioides* filter prevalence 7-day prior to dust storms [%(n/total, 95% CI)]	Proportions test p-value
All events	14	5 (6/115, 2–11)	6 (25/396, 4–9)	0.83
All NOAA events	13	5 (5/107, 2–11)	6 (24/363, 4–9)	0.61
All verified	10	5 (4/85, 2–12)	6 (20/315, 4–9)	0.75

All confidence intervals and proportions tests were computed using Wilson’s score.

### Weather covariate analysis

**Figure 2:**
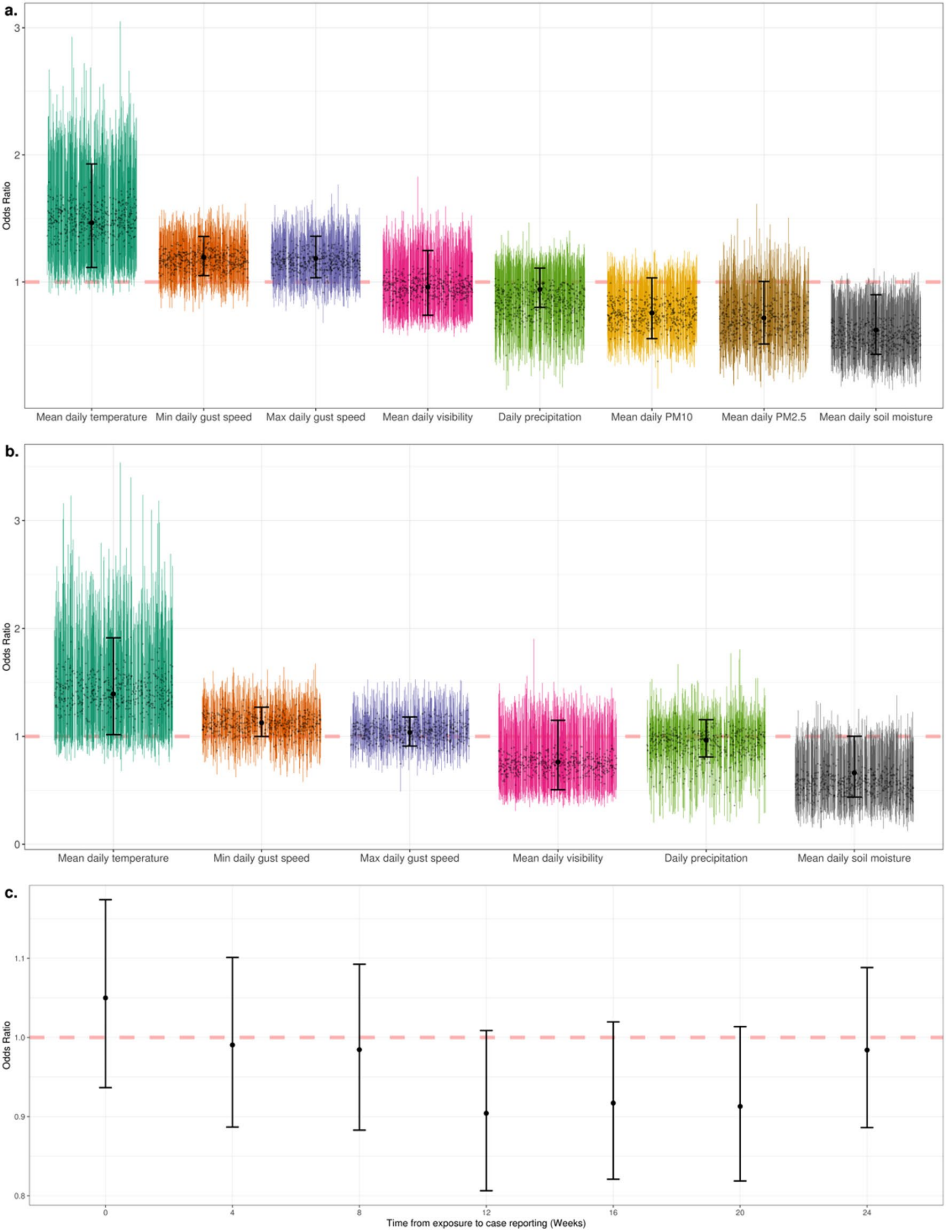
(**a**) Effect of daily environmental measures on filter prevalence across the study period using univariate models. The large black point represents the point estimate of the odds ratio with the error bar representing a 95% confidence interval estimate of the odds ratio. To account for possible influential points, the smaller colored bars represent 500 bootstrapped iterations of the odds ratio estimates and confidence intervals. (**b**) Effect of daily environmental measures on filter prevalence across the study period using multivariable models. The large black point represents the point estimate of the odds ratio with the error bar representing a 95% confidence interval estimate of the odds ratio. The smaller colored bars represent 500 bootstrapped iterations of the odds ratio estimates and confidence intervals. **c.** Effect of weekly *Coccidioides* filter prevalence on reported Valley fever cases across several incubation periods (0–24 weeks) in the greater Phoenix metropolitan area. The point represents the point estimate of the effect with the error bar representing the 95% confidence interval.

Mean daily temperature had the highest positive association with *Coccidioides* detection, with odds ratio (OR) of 1.5 (95% CI 1.1–1.9) in the univariate model and 1.3 (95% CI 1.0–1.9) in the full model (Fig. 2a and b). Further bootstrapping this analysis yielded an OR estimate of 1.5 (95% CI 1.3–1.7) in the univariate model and 1.4 (95% CI 1.1–1.7) in the full model. Lower soil moisture was associated with a higher likelihood of detection, with a OR estimate of 0.6 (95% CI 0.4–0.9), while bootstrapping produced an OR estimate of 0.6 (95% CI 0.4–0.7) in the univariate model. In the multivariable model, moisture availability had a OR estimate of 0.7 (95% CI 0.4–1.0), and OR = 0.6 (95% CI 0.4–0.8) in the bootstrapped model. In addition to temperature and soil moisture, a positive trend between high wind gusts and *Coccidioides* presence was observed. However, this relationship was not significant in all bootstrapped sample sets and produced a OR estimate of 1.2 (95% CI 1.0–1.3) in the univariate and 1.05 (95% CI 0.9–1.2) in the full model. The trend for with minimum gust speed was similar to that of maximum gust speed, with not all bootstrapped sample sets recording a significant effect and OR estimates of 1.2 (95% CI 1.0–1.3) and 1.1 (95% CI 0.9–1.26) in the univariate and full model. PM_2.5_ (Univariate: 0.7, CI 0.5–1.0) and PM_10_ (Univariate: 0.8, CI 0.6–1.0) were slightly negatively associated with detection, while bootstrapping produced estimates of 0.7 (95% CI 0.5–0.9) and 0.8 (95% CI 0.6–0.9). Visibility (Univariate: OR = 1.0, CI 0.7–1.2; Multivariable: OR = 0.76, CI 0.5–1.1) and daily precipitation (Univariate: OR = 0.9, CI 0.8–1.1; Multivariable: OR = 1.0, CI 0.8–1.2) were not statistically associated with *Coccidioides* detection with the collected data. Bootstrapped sampling of the full model did provide a OR estimate of 0.7 (95% CI 0.6–0.9) in the full model for visibility, however this was not consistent within the univariate model (OR = 1, 95% CI 0.8–1.1).

In addition to odds ratios within the univariate and multivariate models, we analyzed the random effects across sites using mixed effects binomial models. The effects of the covariates were generally consistent among sites in temperature and soil moisture, although some sites had variation. For example, one site, “1D”, demonstrated a strong positive association between *Coccidioides* detection and soil moisture, while the other ten sites showed either a negative or no association (Supplemental Fig. 2). To further investigate the effect of season, we did incorporate it into our weather covariate analysis, however, the effect of season had an odds ratio that overlapped 1 and inconsistent effects across sites (Supplemental Analysis 1).

### Land cover analysis

**Figure 3:**
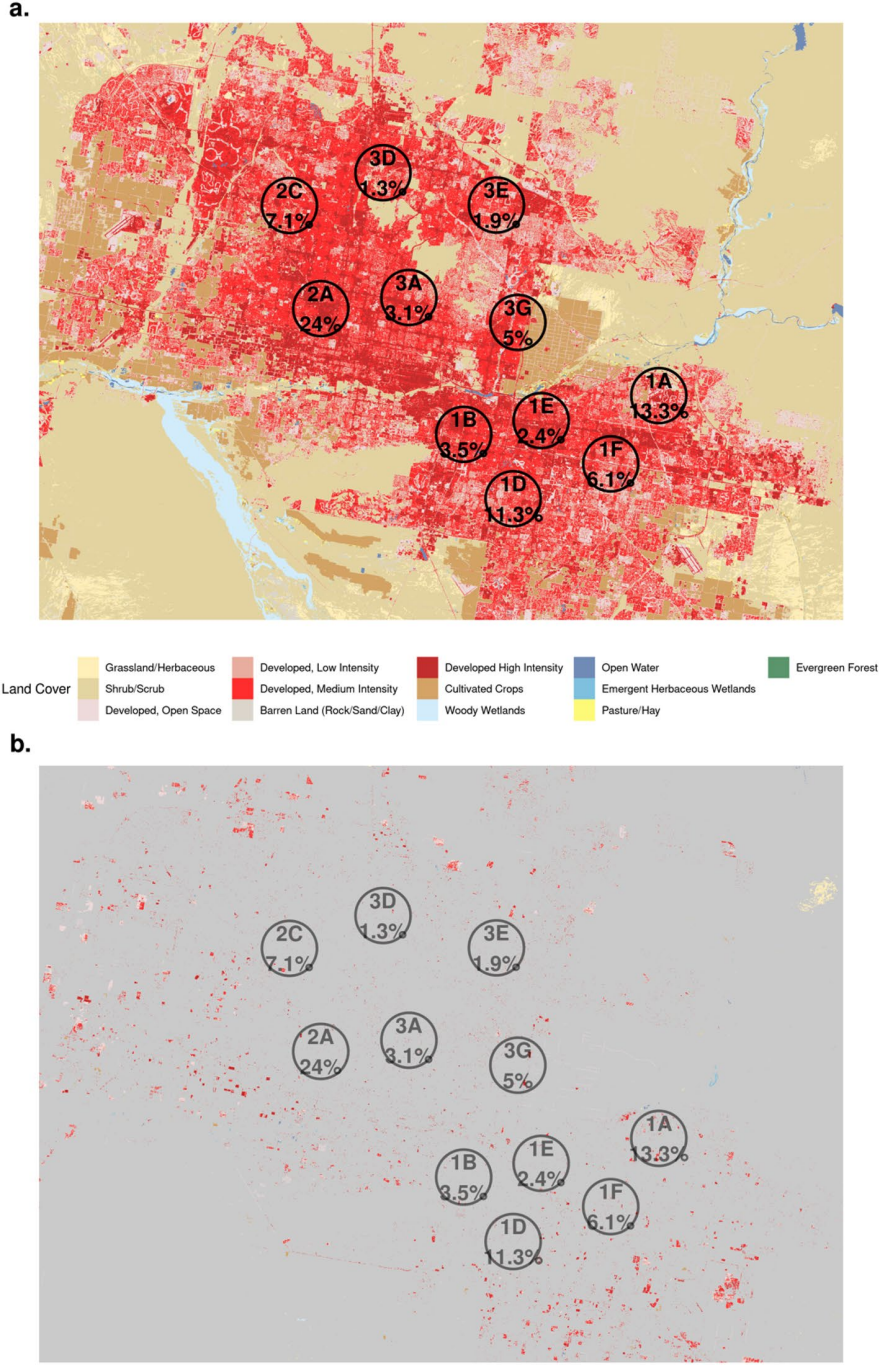
(**a**) Land cover classification across the Phoenix metropolitan area, AZ in 2019, along with the fixed filter sites and aggregated *Coccidioides* prevalence across all filters. (**b**) Land cover change that occurred between 2016 and 2019 in the Phoenix metropolitan area, AZ. The gray areas indicate no change in land cover, with colors representing the updated (2019) land cover classification. Circles represent the fixed filter sites with the aggregated *Coccidioides* prevalence across all filters. Maps were created in R (4.2.0)^30^ using Rstudio editor (2022.02.2)^31^.

Across all sites, medium intensity developed land accounted for most of the land cover, with a mean of 48.4% (Range: 28.8–57.9%, Median: 49.9%) (Fig. 3a). The next most common land cover types were low intensity developed land (Range: 9.7–35.0%, Median: 21.6% Mean: 21.4%) and high intensity developed land (Range: 7.3–40.4%, Median: 15.1%, Mean: 19.6%). Additional land cover classifications included developed open space, cultivated crops, shrub/scrub, and other. *Coccidioides* aggregated filter prevalence was not associated with land cover composition (p = 0.51). Changes of land cover type between 2016 and 2019 entailed urbanization in areas surrounding the Phoenix metropolitan area (Fig. 3b). At filter collection sites, the proportion of area within a 2.4 km radius that underwent land cover turnover ranged from 0.3% (2A) to 3.9% (1A) across study sites with an average of 1.1% (median: 0.7%). The largest changes across sites were the result of increasing developed medium and high intensity areas. *Coccidioides* filter prevalence was not associated with these land cover changes within a 2.4 km radius (p = 0.99).

### Reported Valley fever cases across the study period

Qualitatively, reported Valley fever cases across five regions of the greater Phoenix metropolitan area were generally stable across time and space, with exception of the previously identified^34^ spike at the end of 2017 (Fig. 1b). Utilizing univariate models, detection of *Coccidioides* arthroconidia on the filters was not associated with metropolitan wide clinical cases at any lag periods (period from theoretical exposure to case report), with all confidence intervals overlapping 1.0 (Fig. 2c).

## Discussion

Understanding Valley fever risk has been a research aim of the public health and clinical communities for many years, with the majority of previous work focusing on reported clinical cases to identify risks associated with environmental variables (Table 1). Although these studies have been useful for recognizing possible associations, utilizing case reports introduces several biases and challenges, including highly variable incubation periods among people infected with Valley fever, inconsistent testing, incomplete case finding, and variably lagged diagnosis and reporting. Air filter surveillance for *Coccidioides* allows for direct monitoring of the pathogen within the air and is not subject to these biases, allowing for higher resolution analysis of environmental factors associated with exposure risk. Previously, we developed an improved methodology for detection of *Coccidioides* arthroconidia in the ambient air using daily collected air filters from fixed high-volume sampling and reported the preliminary results of a 45-day air-surveillance from 21 sites in the Phoenix, Arizona, metropolitan area^17^. Here we show results from over 600 days of air surveillance across 11 sites in the same area. To our knowledge, these results represent the largest spatial and temporal airborne survey to date, which was able to identify environmental factors associated with the presence of arthroconidia, explore previously hypothesized aerosolization drivers (e.g., dust storms), as well as unveil notable temporal and spatial dynamics of *Coccidioides* in the air.

Our results demonstrated a highly variable prevalence of the airborne arthroconidia across space and time within an endemic and highly urbanized metropolitan region. Across our 18-month surveillance period, *Coccidioides* prevalence peaked in the spring; however, this was not consistent across sites and driven by increased prevalence at four sites (Sites: 1B, 1D, 2A, and 2C). Additionally, a few sites maintained high daily prevalence of arthroconidia for weeks, while the nearby sites located < 8 km away showed little to no detectable *Coccidioides* during the same time. Furthermore, the periods of high and low daily prevalence of arthroconidia occurred at different times at different sites. The observed heterogeneity among sites is consistent with our understanding of *Coccidioides* lifecycle. For example, it is well documented that *Coccidioides* is not evenly distributed in the soil across space^9^, thus, it not surprising that airborne arthroconidia from disturbed soil will also be unevenly dispersed. This provides evidence that aerosolized *Coccidioides* arthroconidia are not uniformly distributed across an endemic region, nor is the localized geographic risk of exposure.

We hypothesized that local land cover and land cover change (i.e., development) have a large impact on the variability in *Coccidioides* airborne prevalence; however, our land cover analysis did not show a relationship between specific land cover types or land cover change and site level *Coccidioides* prevalence. We expect that this is likely due to a lack of spatial resolution within our analysis, a result of not having exact locations of filter collections. For example, our analysis utilized land cover from a 2.4 km radius around the approximate site location. However, we suspect that sustained high prevalence periods are the result of extremely focal factors, such as localized soil disturbance from construction, which would not have been detected by our analysis.

Although the distribution of arthroconidia across space and time was uneven and often seemingly stochastic, several weather variables were associated with *Coccidioides* detection consistent with the established ecology of *Coccidioides*. For example, mean daily temperature and maximum daily wind gust speeds were both positively associated with *Coccidioides* detection. In addition, mean daily soil moisture was negatively associated with *Coccidioides* detection at most sites, suggesting that warmer days with higher gusts and low soil moisture increase the probability for aerosolization of arthroconidia and *Coccidioides* detection within the air. Although our program covers ~ 18-months of sustained daily air filter and weather variable surveillance, the lack of multi-year surveillance limits the ability to larger-scale incorporate temporally lagged weather variables into our analysis. Previously, temporally lagged weather variables have been identified as significant factors influencing the number of reported Valley fever cases (Table 1); however, to compare these findings to air surveillance, the air surveillance would need to cover numerous consecutive years as well.

In contrast with the weather variable relationships, no increase in *Coccidioides* detection was seen on days with reported dust storms. The possible relationship between *Coccidioides* exposure and significant dust storms (‘haboobs’) has been a subject of recent debate^21,23,35^. Our analysis included dust storm reports through the National Oceanic & Atmospheric Administration Storm Events Database, which relies on several sources including trained spotters, storm chasers, government employees, and public observations. The reliability of these data has been questioned since this dataset is not independently verified^35^; therefore, to improve confidence in the reported dust storm events, we used online images, videos, and news articles to verify reported dust storm instances, improving the reliably of these reports (Supplemental Table 2). In addition, analysis included several subsets of dust storms including all reported events, all NOAA events, and all investigator verified events. Overall, this analysis provides evidence that the prevalence of airborne *Coccidioides* did not increase around the days when dust storms were recorded, consistent with our previous work^17^. However, we caution that this does not preclude the possibility that occasional dust storms in areas with high densities of localized arthroconidia and recent soil disturbance could contribute to airborne arthroconidia and Valley fever cases, as seen in natural disasters^34,36^. Additionally, our analysis did not consider spatial or temporal scales of the dust storm, which were unavailable due to the lack of consistent dust storm reporting.

From an epidemiological perspective, clinical cases did not obviously increase with airborne *Coccidioides* prevalence in localized regions across the Phoenix metropolitan area (Fig. 1b). An increase would have been expected if a few high-risk locales were responsible for most Valley fever cases across space. Instead, Valley fever cases were fairly homogenous across the greater Phoenix metropolitan area, which is dominated by developed urban areas. Although these observations seem to contradict the air surveillance data, they likely result from different spatial and temporal resolutions within the data. Reported case data were available at a coarse scale (community or city). However, even finer spatial scale case data (e.g., patient’s residence) may not reflect the location of exposure. Unlike case data, air filter data represent a snapshot of risk for a small spatial and temporal area. In addition, movement of individuals across space and time was not incorporated into these analyses, thus, it is possible that individuals’ movements from low prevalent sites to higher prevalent sites, or vice-versa, across space and time account for the evenness in reported Valley fever cases. For example, a recent community shed analysis of the Phoenix metropolitan area found that the average commute time was 26 min over a substantial distance (i.e., > five miles*)*^37^, demonstrating extensive daily movement of a large segment of the population across the Phoenix metropolitan area.

The complicated relationship between airborne arthroconidia and reported cases of Valley fever in the Phoenix metropolitan area is further illustrated by the lack in association between *Coccidioides* filter detection and clinical cases, even accounting for variable incubation, testing and reporting periods (Fig. 2c). Our study, though the largest investigating the prevalence of aerosolized arthroconidia to date, used air sampling data from only 11 sites across the greater Phoenix metropolitan area, while reported clinical cases are likely the result of exposure across the entire greater Phoenix metropolitan and surrounding areas, a much larger spatial area. In addition to these spatial scale challenges, case reports introduce additional biases that include diagnostic delays and significant under-diagnosing and under-testing. Though our analysis of temporal lags suggested no relationship between *Coccidioides* detection and clinical cases, we caution that based on the ecology, epidemiology, and public health investigations of *Coccidioides* this is likely not a sufficient conclusion. We hypothesize that if filter surveillance were expanded to provide a complete high-resolution view of *Coccidioides* risk across the greater Phoenix metropolitan area, and clinical testing data were available at finer spatial scale, including epidemiologic data identifying likely exposures sites and timepoints, this relationship would be positive.

Surveillance for airborne *Coccidioides* at fixed locations provides a novel method to assess Valley fever risk, with each filter collecting a total air volume equivalent to that inhaled by ~ 17 people, increasing the likelihood of *Coccidioides* detection compared to lower volume collections. Although further work is needed to identify the public health implications of these data, high resolution temporal and spatial airborne *Coccidioides* prevalence data can help identify environmental factors (e.g., wind, temperature, and soil moisture) that impact the likelihood of arthroconidia aerosolization. In the future, we aim to further understand the differences in *Coccidioides* prevalence across sites by incorporating higher resolution land cover and soil disturbance analyses. In addition, further investigation should be conducted to better characterize how generalizable aerosolized *Coccidioides* prevalence is across small spatial scales; assess the scope of airborne risk following specific contaminated soil disturbance (i.e., plume modeling); and to better understand to what extent a single sampling location (e.g., at a property, block, or neighborhood level) can be used to assess the local risk.

## Conclusions

We demonstrate the ability to study an environmental pathogen in the ambient airspace, a previously underexplored realm, where they may be most affected by changing environmental variables and where they cause human infection. Utilizing fixed air filter units, the presence of airborne *Coccidioides* was detected and measured across a large geographic space for a sustained period of time, providing insights on the aerobiology and exposure risk in a highly endemic metropolitan region. These results demonstrate high spatial and temporal variability in the prevalence of arthroconidia detected on filters positioned at sites across such a region. This variation was seen both within a site and between closely positioned sites suggesting that, like *Coccidioides* presence within the soil, *Coccidioides* presence within the air is highly localized both in time and space. Future studies should prioritize assessing how localized these patterns are and should involve matched air and soil analyses. In addition, no difference in prevalence was seen between days with dust storms when compared to days without dust storms. Covariate analyses revealed that increased filter prevalence rates were observed on days with higher temperatures, higher maximum gust speeds, and lower soil moisture indices, suggesting that these variables are important for arthroconidia aerosolization, and should be considered as weather covariates in future models and analyses. From this study, it can be surmised that *Coccidioides* exposure risk in an endemic area may be most dependent on very localized soil disturbance events (e.g., neighborhood-level site development), that in turn may be enhanced by local or even regional weather features (e.g., wind and aridity). Public health policy will be better informed by understanding such local drivers of increased risk of coccidioidomycosis.

## Methods

### Air sampling

Air sampling was conducted at 11 fixed sampling locations in a ~ 1500 km^2^ region of the Phoenix, Arizona, metropolitan area using portable air sampling units located between 4.5 and 15 feet above the ground, as previously described^17^. The collections are part of ongoing surveillance program conducted by Maricopa County Department of Air Quality, Maricopa County Department of Public Health, and Arizona Department of Health Services. The exact locations of the sites are confidential; thus, approximated locations (~ 2.4 km radius) were used in all analyses; however, generally these air filters are in urbanized regions. Air samples were collected over a 24-h period from ~ 7 a.m. to ~ 7 a.m. The collection device utilized an airflow rate of 100 L/min which is equivalent to the same amount of air that ~ 17 people would breathe in a minute. Hydrophobic polytetrafluoroethylene filters fluorophore membrane filters (3 μm pore size, 47 mm diameter, 150 μm thickness, and 85% porosity, PTFE, EMD Milipore, Danvers, MA, USA) were used for all collections. Air sampling was conducted over three time periods. The first two periods (August 24–26, 2016 and September 25–November 8, 2016) served as a pilot program for a larger surveillance program and data from the pilot program were previously published^17^. The larger program was initiated after the pilot program and collected samples from the beginning of January, 2018 through the end of July, 2019. Sample processing was split between two laboratories: (1) Lab A, at the Translational Genomics Research Institute; and Lab B, at the Centers for Disease Control and Prevention. Since there were slight differences in equipment availability between each lab, test sensitivity was verified across labs by exchanging *Coccidioides* spiked filters and a dilution series of *Coccidioides* DNA.

### DNA isolation

Dneasy PowerLyzer PowerSoil kit (Qiagen) was used for extraction of genomic DNA from filters in accordance with the manufacturer’s instructions and the following modifications; filters were sectioned into ~ 1 cm pieces using sterile scissors, loaded directly into the PowerBead tubes provided in the kit. At lab A, homogenization was performed using MP BioMedicals FastPrep-24™ Classic benchtop bead beating lysis system for seven 1-min cycles at 6.0 m/s with 5 min cool down breaks between each cycle. At lab B, homogenization was performed using Qiagen PowerLyzer24 benchtop bead beating lysis system for seven 1-min cycles at 3700 rpm with 5-min cool down breaks between each cycle. Upon addition of elution buffer, a 5-min incubation was completed prior to spinning the filter. DNA was eluted into 50 µL of Qiagen’s C6 solution.

### *Coccidioides* detection

A single-tube nested real-time PCR assay^38^ which is based on the CocciEnv real-time PCR target^39^ was run in duplicate to analyze DNA samples for presence of *Coccidioides* DNA*.* At lab A, each 10 µL reaction mixture contained 2 µL DNA template, 5 µL 2× SSOAdvanced Universal Probes Supermix (Bio-rad), 240 nM each of primers and probe, and 2.5 µL nuclease-free water. Thermocycling conditions consisted of an initial denaturation for 10 min at 95 °C, followed by 11 outer amplification cycles at 95 °C 10 s, 65 °C 30 s, 72 °C 15 s, and 45 inner amplification cycles at 95 °C 10 s, 52 °C 30 s, 72 °C 15 s on a Biorad CFX Connect. At lab B, the assay was run in 20 µL reactions containing TaqMan Universal Polymerase Chain Reaction (PCR) Master Mix (Applied Biosystems, Grand Island, NY, USA), 240 nM each of primers and probe, BSA (2 ng/μl; BSA) and 2 μl DNA template. Thermocycling conditions consisted of an initial denaturation for 10 min at 95 °C, followed by 25 outer amplification cycles at 95 °C 10 s, 65 °C 30 s, 72 °C 15 s, and 45 inner amplification cycles at 95 °C 10 s, 52 °C 30 s, 72 °C 15 s on a Rotor-Gene 6000 thermocycler (Qiagen; Valencia, CA, USA). Samples were considered positive if they displayed a Ct less than 45 and displayed logarithmic amplification plots on at least one of the duplicate real-time PCR reactions.

### General analyses

All analyses were conducted in R (4.2.0)^30^ using the Rstudio editor (2022.02.2)^31^ using tidyverse^40^, ggmap^32^, DirichletReg^41^, sp^42^, and lme4^43^ packages. Filter prevalence was calculated by dividing positive filters by the total filters. We used Wilson’s score to generate 95% binomial confidence intervals for filter prevalence measurement error^30,44^.

### Dust storm records

Dust storm records were collected from NOAA’s Storm Events Database for Maricopa County and across the study period^45^, which relies on trained spotters, storm chasers, government employees and public observations. Events were included if they were reported in Maricopa County, AZ and were reported as “Dust Storm”. Since the database is not independently verified and no information on the scale of the events is available, to further increase confidence in the reported events, reports were verified by utilizing Google searching “dust storm Phoenix AZ” along with the year and/or the date of the event. We counted an event as verified if we found a news story, social media post with photos or videos, or other documentation verifying the event occurrence. A total of 26 dust storms were reported to the NOAA database, of which, 20 (77%) could be verified with online reports. Six of the 26 (23%) were not verified with the online search method, however, these events were still included in the analysis. An additional six dust storm events were identified via internet searching during the time period of interest and were included in the “All Events” analysis (Supplemental Table 2). In addition to the researcher verification, we investigated the relationship of PM_10_ and PM_2.5_ to reported dust storms. Generally, days with reported dust storms had higher PM_10_ and PM_2.5_ values. For example, days with reported and verified dust storms had a mean PM_2.5_ value of 11.6 (95% CI 9.4–13.7) compared to 7.58 (95% CI 7.4–7.78) on days without reported dust storms. Similarly, for days with reported and verified dust storms PM_10_ values had an average of 63.1 (95% CI 48.4–77.8) compared to 24.4 (95% CI 23.9–25) on days without reported dust storms. However, this distribution overlaps with some of the verified and unverified dust storm days having near “normal” PM values (Supplemental Fig. 3). For an initial analysis, *Coccidioides* detection and dust storm presence was compared across all available data and compared *Coccidioides* prevalence on days with a reported dust storm to days without. Further analyses were limited to 2018, where the most consistent filter surveillance data were available allowing prevalence to be calculated in the 7-days prior to the dust storm and on days with reported dust storms. Analysis utilizing filter data and dust storm events prior to 2018 have been previously published^17^. All statistical comparisons were performed with Wilson’s score accessed through prop.test^30,44^.

### Weather data

Hourly weather predictions were extracted from the NOAA’s High Resolution Rapid Refresh (HRRR) model^46,47^. HRRR model was used since local weather station data from each air filter site was not available and we were not able to quantify the exact distance from the nearest weather station to the air filter collections site. Archived HRRR data for the study period were downloaded from Amazon Web Services (https://registry.opendata.aws/noaa-hrrr-pds/). The HRRR model provides hourly predictions for a variety of weather variables (including wind speed, temperature, visibility, precipitation, and surface soil moisture) for the continental United States at a gridded 3-km spatial resolution and the 1-h forecast was used for all analyses. Since the exact location of the filter collection locations were confidential and not available, data were extracted from 2.4 km radius around the approximate filter collection location and a weighted average was used to average the gridded values from the buffer zone. For each day (8 a.m.–7 a.m.) and site, summary statistics (sum (precipitation), mean, median, min, and max) were calculated for each variable. Daily observations were removed (n = 1, 2019-03-09) if they had less than 18 hourly observations within a single day.

To validate HRRR model data, hourly summarized weather station observations from the Flood Control District of Maricopa County weather stations (n = 39) were compared to the hourly HRRR model forecasts across temperature and max wind gust (unpublished data and analyses). Temperature was highly correlated across the sites with an average Pearson correlation of 0.89 (Min: 0.81, Max: 0.93). HRRR gust speeds were moderately correlated with weather station observations with a mean site level Pearson correlation of 0.54 (Min: 0.29, Max: 0.72). Other included HRRR model variables were not available for comparison within the weather station dataset.

### Particulate data

Particulate data were accessed through U.S. Environmental Protection Agency’s Air Monitoring archives^48^. In total, PM_10_ (particulate matter < 10 microns) and PM_2.5_ (particulate matter < 2.5 microns) values were collected from a total of 18 air quality monitoring sites in the Phoenix metropolitan area. Filter collection sites were then paired with to closest PM_10_ monitor location up to a 4.8 km radius. This technique provided data for five of the 11 sites in this analysis (“1B,” “1D,” “1E,” “2A,” and “2C”). In addition, PM_2.5_ data were collected from four sites in this analysis (“1B,” “1E,” “2A”, and “2C”).

### Environmental covariate analysis

Prior to the analysis, available hourly weather covariates (including temperature, wind speed, visibility, precipitation and particulate matter) were summarized at the daily level, using max, min, and mean, and were grouped into categories (i.e., wind/gust speed, temperature, visibility, soil moisture, precipitation, PM_10_ and PM_2.5_) and collinearity within groups was analyzed to reduce the dimensionality of the analysis. Due to the high amount of collinearity within each category (i.e., min temperature, mean temperature, and max temperature), a primary variable from each category was retained if it was expected to more directly influence the aerobiology of *Coccidioides* (i.e., daily max gust speed and daily accumulated precipitation), otherwise, the mean of the category was selected. Once the primary variable was selected, other variables were removed from consideration if they had a correlation greater than 0.6 to the selected variable. All predictor variables were centered and scaled prior to statistical modeling to improve model convergence and interpretation.

Environmental covariate analyses (temperature, wind speed, visibility, precipitation and particulate matter) were conducted using generalized linear mixed models, using the logit link function and binomial distribution (using “lme4” package^43^), to explain the daily proportion of filter positives using both univariate and multivariable models. Site was included as a random effect on both the slope and the intercept, allowing each site to have an independent calculated slope and intercept to identify if effects were consistent across sites. Multivariable models excluded PM_10_ and PM_2.5_ variables as these variables were not available for all sites (see “Particulate data”). Within the full multivariable model, the most parsimonious random effects model structure was selected by calculating all possible combinations of random effects on predictor variables and selecting the model based on AIC. In all iterations, models including random effects for both site and all covariate slopes were the most parsimonious and used in further analyses.

Models were fit using bound optimization by quadratic approximation optimizer and up to 20,000 function evaluations. Confidence intervals around fixed effects were generated using Wald confidence intervals. To explore the stability of odd ratio estimates and account for possibility of influential observations, models were built using the full dataset and 500 bootstrapped iterations. Multicollinearity within the full model was assessed with variance inflation factor (VIF), to ensure all variables in the model were uncorrelated. An overall effect, odds ratio (OR), was recorded from each mixed effect model and used to identify covariates with generally positive (OR > 1) or negative (OR < 1) associations with *Coccidioides* prevalence on filters. In addition to the average effect of each covariate across sites, ORs were calculated for each site to analyze the consistency of the effects across sites.

Univariate model structure:

$${\text{logit}}\left(P\left(Positive\, Filter\right)\right)={(\beta }_{0}\text{+}{S}_{0\, Site})+{(\beta }_{1}+{S}_{\,1Site})\text{Single\, Predictor + }{\mathcal{E}}_{Site\, i}$$

Multivariable model structure:

$$\begin{aligned} {\text{logit}}\left(P\left(Positive\, Filter\right)\right) & ={(\beta }_{0}\text{+}{S}_{0 \,Site})+{(\beta }_{1}+{S}_{1\,Site})\text{Max Gust Speed } + { (\beta }_{2}+{S}_{2\, Site})\text{Max Gust Speed } \\ & \quad + { (\beta }_{3}+{S}_{3\, Site})\text{Min Gust Speed } + { (\beta }_{4}+{S}_{4\, Site})\mathrm{Mean \; Temperature\,}{+ \,(\beta }_{5}+{S}_{\,5 Site})\mathrm{Mean \; Visibility } \\ & \quad+ \text{ } {(\beta }_{6}+{S}_{6\, Site})\text{Mean Soil Moisture } + { (\beta }_{7}+{S}_{7\, Site})\mathrm{Total \; Precipitation }+ \text{ } {\mathcal{E}}_{Site\, i}\end{aligned}$$

### Landcover analysis

Land cover for the Phoenix metropolitan area was acquired from the National Land Cover Database for 2019 and 2016 and was at a 30-m resolution^49,50^. Using the 2019 NLCD product, the proportion of each land cover class was extracted for each site with a 2.4 km radius buffer around the approximate site location. Land cover classes with a proportion of less than 1% across sites (Grassland/Herbaceous, Open Water, Woody Wetlands, Emergent Wetlands, Barren Lands and Pasture/Hay) were grouped into a single group (“Other”). In addition to 2019 land cover dataset, land cover change between 2016 and 2019 was calculated at the pixel level using the NLCD 2016 and NLCD 2019 product. The effect of land cover on site level *Coccidioides* prevalence was analyzed using Dirichlet regression^51^. Dirichlet models summarize the proportions of land coverage or change by site as a response vector (with a sum to one constraint), with either an intercept only model (null model) or *Coccidioides* prevalence as a predictor variable (experimental model). A statistically significant association was identified by measuring the comparing the models using the difference between squared deviance and chi-squared test statistic.

### Maricopa County case data

Reported Maricopa County Valley fever case data were acquired from the Arizona Department of Health Services and TGen's Office of Research Compliance and Quality Management determined that the research met the criteria for IRB exemption defined at 45 CFR 46.104(d)(4). To investigate possible differences in cases across space and time, reported cases were filtered to the greater Phoenix metropolitan area and grouped into four categories based on the location of the reported city, which were not independently verified. For each analysis the earliest date reported (generally, the date of the first positive test) of each case was used. Spatially, the West Valley included cases with reported cities that were west of Interstate 17. Central Valley included the City of Phoenix proper. East Valley included cities east of State Route 51 and North of Loop 202. The South East Valley was considered areas south of the northern section of Loop 202 and east of the western section of Loop 202. Data were normalized across these regions by using a 3-week rolling lagged mean and dividing that by mean weekly cases to allow all data to be visualized at a single scale.

### Univariate Valley fever case analysis

A generalized linear model, using the logit link function and binomial distribution was used to compare the relationship of total weekly (3-week rolling average) reported Valley fever cases across the greater Phoenix metropolitan area to aggregated *Coccidioides* filter prevalence across weeks. Weeks that had less than 20 filters collected were removed from this analysis. To account for the difficulties in Valley fever diagnosis and possible delays in reporting, a total of seven different lag periods, time between filter prevalence and case report, were analyzed including 0, 4, 8, 12, 16, 20, and 24 weeks. Point estimates and 95% confidence intervals of the odds ratios were then computed and compared across the expected lag periods.

Univariate Model Structure:

$${\text{logit}}\left(\frac{Positive \; Filters}{Negative \; Filters} \right)={\beta }_{0}+({\beta }_{1}\cdot \text{Laged Cases)}$$

### Supplementary Information

Supplementary Information 1.

Supplementary Information 2.

## Data Availability

*Coccidioides* presence/absence data are available as a supplemental file. Reported Arizona Valley fever case counts are available from Arizona Department of Health Services. All other data sources in this analysis previously publicly available.
