# Supplementary material for: Understanding the exposure risk of aerosolized Coccidioides in a Valley fever endemic metropolis
Source: Sci Rep. 2024 Jan 15;14:1311. doi: 10.1038/s41598-024-51407-x (PMC10789871; doi:10.1038/s41598-024-51407-x)
Supplement: Supplementary file 1 — Supplementary Information 1. [file 41598_2024_51407_MOESM1_ESM.docx]

Understanding the exposure risk of aerosolized *Coccidioides* in a Valley fever endemic metropolis

**Supplemental File 1.** Comma separated sheet of a line list with all available filter data.

**
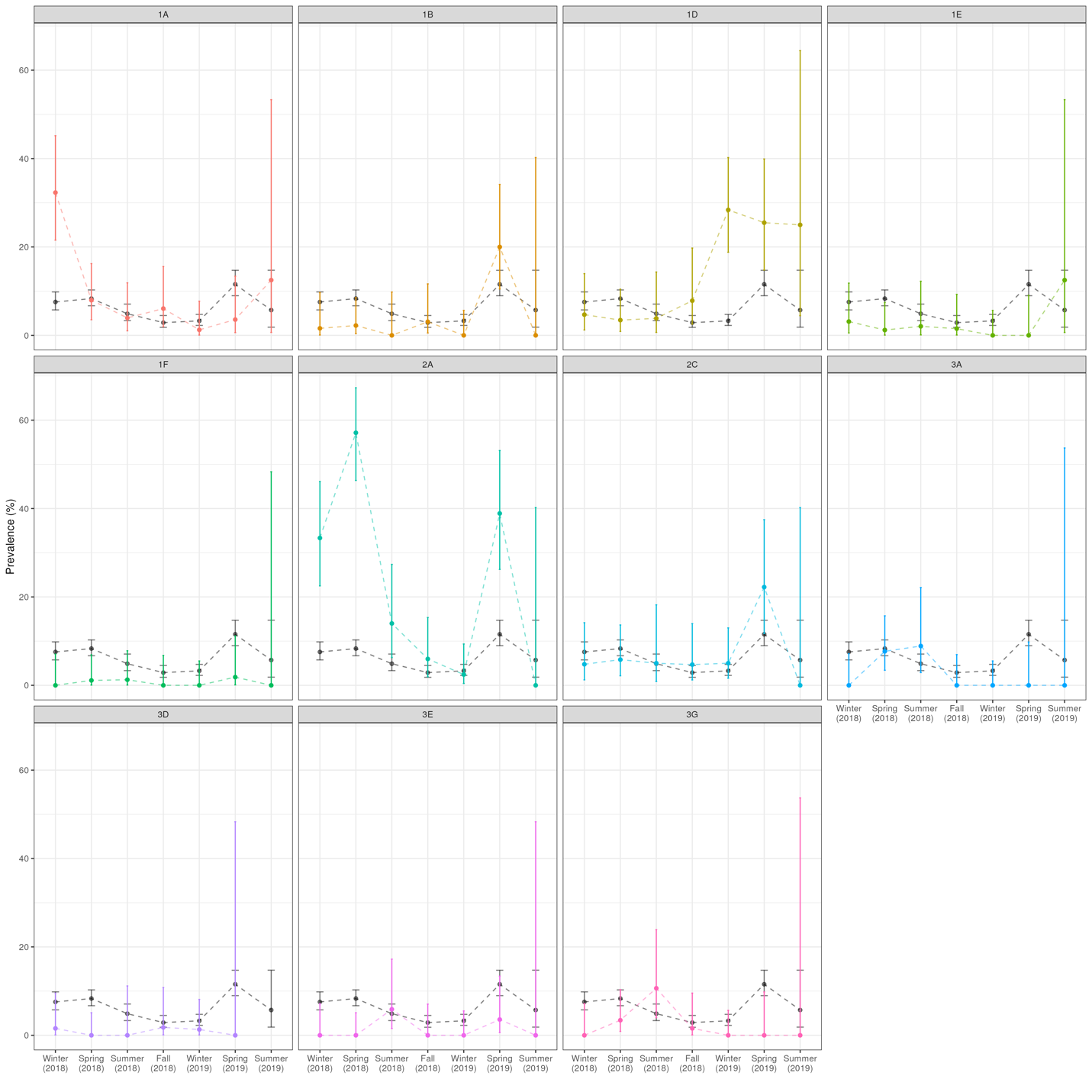
**

**Supplemental Figure 1.** Seasonal *Coccidioides* prevalence (% positive filters) and 95% confidence intervals across sites with the across site average displayed in black.


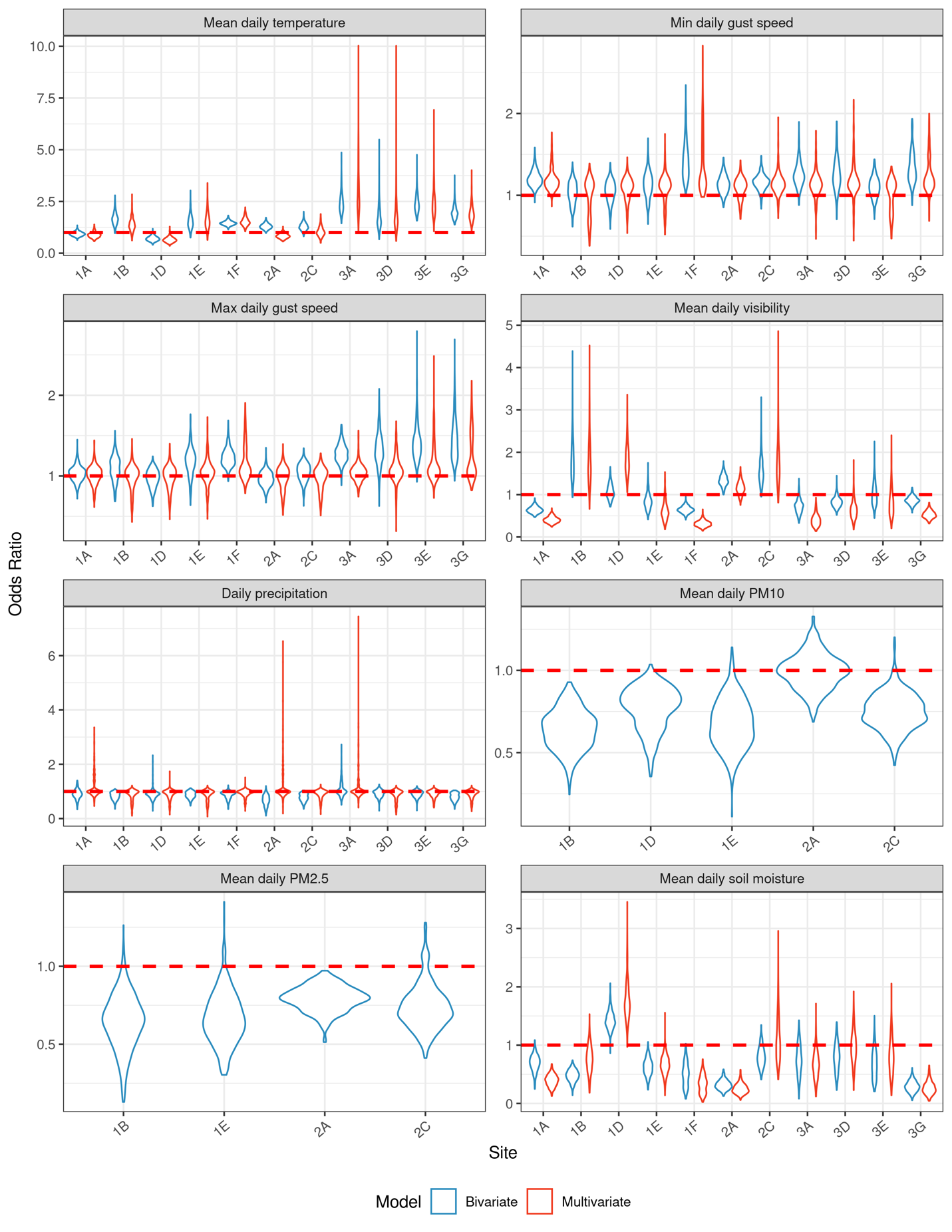


**Supplemental Figure 2.** Violin plot of the distribution of odds ratios of univariate and multivariable random effect models across sites and covariates.


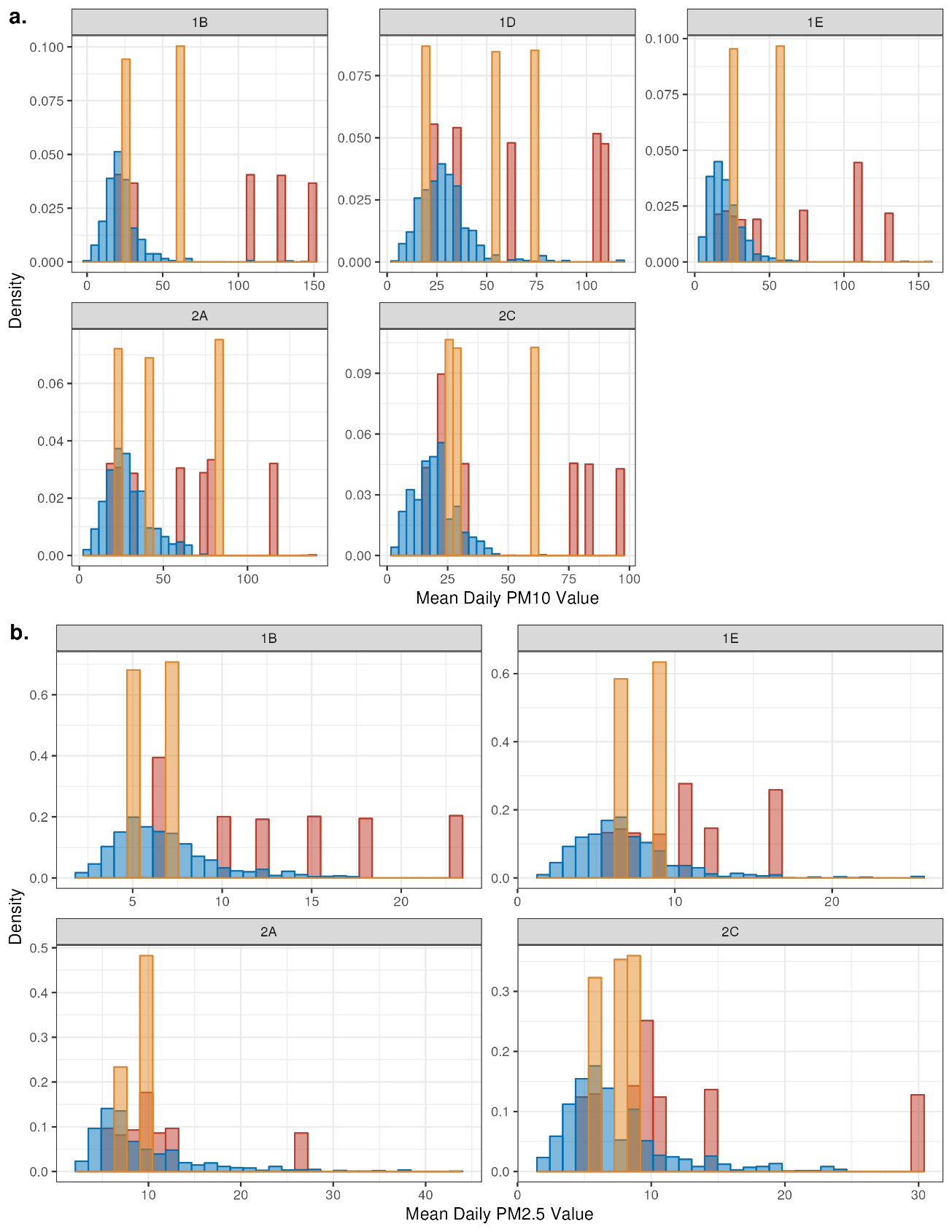


**Supplemental Figure 3.** Distribution of mean daily PM_10_ (**a.**) and PM_2.5_ (**b.**) values across days without reported dust storms (blue), with verified dust storms (red), and with unverified dust storms (orange).

**Supplemental Table 1.** Yearly filter prevalence of *Coccidioides* across sites.

| Site | 2015  [%(n/total, 95% CI)] | 2016  [%(n/total, 95% CI)] | 2017  [%(n/total, 95% CI)] | 2018  [%(n/total, 95% CI)] | 2019  [%(n/total, 95% CI)] |
| --- | --- | --- | --- | --- | --- |
| 1A | 33.3 (1/3, 1.77–87.47) | 55.6 (25/45, 40.12–70.05) | No filters collected | 11.6 (35/303, 8.28–15.83) | 2.9 (4/137, 0.94–7.77) |
| 1B | 33.3 (1/3, 1.77–87.47) | 0 (0/45, 0–9.8) | No filters collected | 1.8 (5/271, 0.68–4.5) | 7.6 (10/132, 3.9–13.85) |
| 1D | 0 (0/3, 0–69) | 2.3 (1/44, 0.12–13.51) | No filters collected | 4.6 (12/259, 2.53–8.16) | 27.9 (36/129, 20.55–36.61) |
| 1E | 50 (1/2, 9.45–90.55) | 4.5 (2/44, 0.79–16.7) | 4.8 (4/83, 1.56–12.55) | 1.9 (5/270, 0.68–4.51) | 0.8 (1/131, 0.04–4.81) |
| 1F | 66.7 (2/3, 12.53–98.23) | 68.2 (30/44, 52.29–80.93) | 0 (0/78, 0–5.85) | 0.6 (2/308, 0.11–2.58) | 0.7 (1/136, 0.04–4.64) |
| 2A | 0 (0/2, 0–80.21) | 8.7 (4/46, 2.82–21.69) | No filters collected | 30.1 (85/282, 24.92–35.92) | 16.8 (23/137, 11.15–24.34) |
| 2C | 50 (1/2, 9.45–90.55) | 4.3 (2/46, 0.76–16.04) | No filters collected | 5 (13/261, 2.79–8.57) | 11.9 (15/126, 7.04–19.18) |
| 3A | 33.3 (1/3, 1.77–87.47) | 4.4 (2/45, 0.77–16.36) | No filters collected | 4 (11/272, 2.14–7.32) | 0 (0/126, 0–3.69) |
| 3D | 66.7 (2/3, 12.53–98.23) | 0 (0/45, 0–9.8) | No filters collected | 0.8 (2/256, 0.14–3.1) | 1.3 (1/76, 0.07–8.11) |
| 3E | 66.7 (2/3, 12.53–98.23) | 2.1 (1/47, 0.11–12.72) | 3.3 (2/61, 0.57–12.36) | 1.1 (3/276, 0.28–3.41) | 1.4 (2/138, 0.25–5.67) |
| 3G | 33.3 (1/3, 1.77–87.47) | 19.1 (9/47, 9.65–33.73) | 8.2 (6/73, 3.39–17.65) | 3.3 (9/270, 1.64–6.45) | 0 (0/124, 0–3.74) |

Supplemental Table 2. NOAA Storm Event’s Database with reported “dust storms” and online reference for verification.

| **NOAA EVENT ID** | **CZ_NAME_STR** | **BEGIN**  **DATE** | **SOURCE** | **Online Source** |
| --- | --- | --- | --- | --- |
| 588992 | GREATER PHOENIX AREA (ZONE) | 8/11/15 | Trained Spotter | <https://www.washingtonpost.com/news/capital-weather-gang/wp/2015/08/11/strong-winds-kick-up-dangerous-dust-storm-in-phoenix-photos/> |
| 592777 | GREATER PHOENIX AREA (ZONE) | 8/25/15 | Amateur Radio | <https://www.washingtonpost.com/news/capital-weather-gang/wp/2015/08/26/photos-mammoth-dust-storm-engulfs-parts-of-phoenix-area/> |
| NA | NA | 9/3/2015 | Not Detected | <https://www.youtube.com/watch?v=ckDSgH_LQtU> |
| 600082 | GREATER PHOENIX AREA (ZONE) | 10/16/15 | Trained Spotter | https://www.youtube.com/watch?v=yrrcGws0dXI https://www.youtube.com/watch?v=n5BbmA0D1gI |
| 643096 | SOUTHWEST MARICOPA COUNTY (ZONE) | 7/1/16 | Trained Spotter | Unverified |
| NA | NA | 7/2/2016 | Not Detected | <https://www.12news.com/article/weather/monsoon/dust-storm-southeast-valley/75-b49378ad-ac27-4fc8-98d3-9ad3dda433f4> |
| 643098 | GREATER PHOENIX AREA (ZONE) | 7/19/16 | Trained Spotter | <https://www.youtube.com/watch?v=r-tL8tVSCQw> |
| 645900 | GREATER PHOENIX AREA (ZONE) | 7/29/16 | Trained Spotter | <https://www.youtube.com/watch?v=NFuFWh8v5D8> |
| 647016 | GREATER PHOENIX AREA (ZONE) | 8/3/16 | Trained Spotter | Unverified |
| 647196 | GREATER PHOENIX AREA (ZONE) | 8/9/16 | Trained Spotter | <https://www.youtube.com/watch?v=WPw6aCF7BlE> |
| 647643 | GREATER PHOENIX AREA (ZONE) | 8/20/16 | Trained Spotter | <https://www.youtube.com/watch?v=b3rf1ZO9qtQ> |
| 647783 | GREATER PHOENIX AREA (ZONE) | 8/21/16 | Trained Spotter | <https://www.youtube.com/watch?v=EsltvDvsgk4> |
| 647937 | GREATER PHOENIX AREA (ZONE) | 8/24/16 | Trained Spotter | <https://www.12news.com/video/weather/dust-storm-blowing-near-scottsdale/75-2340806> |
| NA | NA | 7/7/17 | Not Detected | <https://www.youtube.com/watch?v=C50y9GQeMkY> |
| 708280 | GREATER PHOENIX AREA (ZONE) | 7/14/17 | Public | <https://www.youtube.com/watch?v=9imTAU18tbQ> |
| NA | NA | 7/15/17 | Not Detected | <https://www.youtube.com/watch?v=svQ5YB_1u9k> |
| NA | NA | 7/16/17 | Not Detected | <https://www.azcentral.com/story/news/local/arizona/2017/08/10/dust-storm-causes-fatal-crash-near-arizona-new-mexico-line/557645001/> |
| 714579 | GREATER PHOENIX AREA (ZONE) | 9/7/17 | NWS Employee | <https://www.12news.com/video/weather/what-it-looks-like-inside-an-arizona-dust-storm/75-2723552> |
| 745618 | EAST VALLEY (ZONE) | 4/12/18 | Trained Spotter | <https://www.youtube.com/watch?v=beiU8U6GQTw> |
| 745623 | SOUTHEAST VALLEY/QUEEN CREEK (ZONE) | 4/19/18 | Trained Spotter | Unverified |
| 757079 | SOUTHEAST VALLEY/QUEEN CREEK (ZONE) | 7/5/18 | Trained Spotter | <https://www.youtube.com/watch?v=rYkkHWI9NhE> |
| 765631 | SOUTHEAST VALLEY/QUEEN CREEK (ZONE) | 7/9/18 | Trained Spotter | <https://www.washingtonpost.com/news/capital-weather-gang/wp/2018/07/10/one-of-the-most-incredible-sights-monster-dust-storm-sweeps-across-southern-arizona/> |
| 758500 | SOUTHEAST VALLEY/QUEEN CREEK (ZONE) | 7/10/18 | Trained Spotter | <https://www.youtube.com/watch?v=bI-dO7RI3lE> |
| NA | NA | 7/14/18 | Not Detected | <https://www.azcentral.com/story/news/local/arizona-weather/2018/07/14/rain-hail-hit-cave-creek-phoenix-northern-arizona-flooding-road-closures/785794002/> |
| 762134 | NORTHWEST VALLEY (ZONE) | 7/28/18 | Trained Spotter | Unverified |
| 767085 | CAVE CREEK/NEW RIVER (ZONE) | 7/30/18 | Trained Spotter | https://www.azcentral.com/story/news/local/phoenix-breaking/2018/07/30/dust-storm-moves-into-phoenix-area-thunderstorms-approach-areas/869063002/ |
| 763468 | EAST VALLEY (ZONE) | 8/2/18 | Trained Spotter | https://www.youtube.com/watch?v=KYxe1ZAJAIc |
| 767519 | EAST VALLEY (ZONE) | 8/7/18 | Trained Spotter | <https://www.azcentral.com/story/news/local/phoenix-weather/2018/08/07/monsoon-returns-dust-rain-move-into-phoenix-area/932216002/> |
| 769084 | SCOTTSDALE/PARADISE VALLEY (ZONE) | 8/8/18 | Public | <https://www.youtube.com/watch?v=mU7dUL52Ae8> |
| 769875 | SONORAN DESERT NATL MONUMENT (ZONE) | 8/10/18 | Public | Unverified |
| 771788 | SCOTTSDALE/PARADISE VALLEY (ZONE) | 8/12/18 | Trained Spotter | <https://www.azcentral.com/videos/news/local/phoenix-weather/2018/08/13/dust-storm-halts-traffic-loop-101-august-12-2018/973944002/> |
| 773586 | SOUTHEAST VALLEY/QUEEN CREEK (ZONE) | 9/2/18 | AWOS | Unverified |

**Supplemental Analysis 1. Incorporation of season into the multivariable weather model.**

To further investigate the effect of seasonality on *Coccidioides* aerosolization, we incorporated seasonality into our multivariable model. No season had a significant global effect within this model, with all odds ratios (OR) overlapping one (Supplemental Analysis Table 1). In addition, there was a significant amount of variability of random effects across sites for each season (Spring OR range: 0.1-17.1, Summer OR range: 0.1-3.1, and Winter OR range: 0.1-11.0). Due to the variability across sites and the short 18-month sustained surveillance period, models including seasonality were not used further within this analysis.

Multivariable Model Structure:

$$\text{logit}\left( P\left( Positive Filter \right) \right)={(\beta}_{0}\text{+}S_{0 Site})+{(\beta}_{1}+S_{1Site})\text{Max Gust Speed +}{(\beta}_{2}+S_{2 Site})\text{Max Gust Speed +}{(\beta}_{3}+S_{3 Site})\text{Min Gust Speed +}{(\beta}_{4}+S_{4 Site})Mean Temperature {+ (\beta}_{5}+S_{5 Site})Mean Visibility \text{+ }{(\beta}_{6}+S_{6 Site})\text{Mean Soil Moisture +}{(\beta}_{7}+S_{7 Site})Total Precipitation \text{+ }{(\beta}_{8}+S_{8 Site})\mathrm{Season}\text{+ }\mathcal{E}_{Site i}$$

Supplemental Analysis Table 1. Odds ratios of fixed effects from supplemental analysis multivariable model. Note: Seasonality was included as a factor, and fall was used as the base factor within the seasonal covariate; thus, Spring, Summer, and Winter were compared to Fall.

| **Variable** | **Odds Ratio** |
| --- | --- |
| Mean daily temperature | 1.6 (95% CI: 1.0 – 2.5) |
| Min daily gust speed | 1.2 (95% CI: 1.0 – 1.3) |
| Max daily gust speed | 1.0 (95% CI: 0.9 – 1.2) |
| Mean daily visibility | 0.8 (95% CI: 0.5 – 1.2) |
| Daily precipitation | 0.9 (95% CI: 0.7 – 1.2) |
| Mean daily soil moisture | 0.7 (95% CI: 0.5 – 1.1) |
| Season: Spring | 0.7 (95% CI: 0.2 – 2.4) |
| Season: Summer | 0.8 (95% CI: 0.3 – 2.2) |
| Season: Winter | 0.8 (95% CI: 0.2 – 2.8) |
